# Ethnic disparities attributed to the manifestation in and response to type 2 diabetes: insights from metabolomics

**DOI:** 10.1007/s11306-022-01905-8

**Published:** 2022-06-28

**Authors:** Sampara Vasishta, Kailash Ganesh, Shashikiran Umakanth, Manjunath B Joshi

**Affiliations:** 1 Department of Ageing Research, Manipal School of Life Sciences, Manipal Academy of Higher Education, 576104 Manipal, India; 2 Department of Medicine, Dr. T.M.A. Pai Hospital, 576101 Udupi, India; 3 Manipal School of Life Sciences, Planetarium Complex Manipal Academy of Higher Education Manipal, 576104 Manipal, India

**Keywords:** Type 2 diabetes, Ethnicities, Metabolomics, Diet, Genetics

## Abstract

Type 2 diabetes (T2D) associated health disparities among different ethnicities have long been known. Ethnic variations also exist in T2D related comorbidities including insulin resistance, vascular complications and drug response. Genetic heterogeneity, dietary patterns, nutrient metabolism and gut microbiome composition attribute to ethnic disparities in both manifestation and progression of T2D. These factors differentially regulate the rate of metabolism and metabolic health. Metabolomics studies have indicated significant differences in carbohydrate, lipid and amino acid metabolism among ethnicities. Interestingly, genetic variations regulating lipid and amino acid metabolism might also contribute to inter-ethnic differences in T2D. Comprehensive and comparative metabolomics analysis between ethnicities might help to design personalized dietary regimen and newer therapeutic strategies. In the present review, we explore population based metabolomics data to identify inter-ethnic differences in metabolites and discuss how (a) genetic variations, (b) dietary patterns and (c) microbiome composition may attribute for such differences in T2D.

## Introduction

The convergence of biological/genetic factors associated with geographical origins, culture, economic, political/legal factors and race contributes to the multidimensional nature of ethnic diversity (Williams, 1997). Ethnic differences significantly contribute to susceptibility and responses to various infectious and non-infectious diseases. Health inequalities in diabetes among ethnicities have been known for a long time although ethnicities showing a higher risk for developing diabetes change over time (Hopkins, 2021). An alarming increase in T2D subjects and subsequent vascular complications change quality of life, increase in demand for health services and costs and thereby alter the disease profile of the population (Harding et al., 2019). Genetic makeup along with differences in dietary patterns, socioeconomic status, environment, access to medical facilities and psychosocial factors attribute to ethnic disparities in Type 2 Diabetes (T2D). Multiple studies have also pointed out ethnic inequalities in T2D associated co-morbidities such as insulin resistance, predisposition to vascular complications, the prevalence of risk factors and response to therapeutic strategies (Golden et al., 2012). However, underlying mechanisms responsible for inter-ethnic differences in T2D are poorly understood.

Across the world, South Asians display an increased burden of T2D in comparison with population from non-White and White ethnic backgrounds (Abate & Chandalia, 2001). Nurses’ Health Study (NHS) which recruited non-Hispanic Whites, Asians, Hispanics and black women aimed to formulate the dietary diabetes risk reduction score. This study revealed that risk reduction score was inversely associated with the risk of developing T2D in population from all ethnic backgrounds and a strong association was observed in minority women (Rhee et al., 2015). The Health Improvement Network (THIN) study conducted in the United Kingdom (UK) revealed that Asians (7.69%) and people from Black ethnicities (5.58%) had a high prevalence of T2D when compared to that of other ethnic groups (3.42%) (Pham et al., 2019). A multi-ethnic study in Scotland showed that Indians and Pakistanis with T2D contained suboptimal glycaemic control (Glycated hemoglobin (HbA1c) > 7.5%) in comparison with the White Scottish population (Negandhi et al., 2013). In low- and middle-income countries, an association between the increase in body weight and obesity leading to cardio-metabolic morbidities including diabetes was found (Popkin, 2015). In the mixed population of Canada under the Diabetes Population Risk Tool (DPoRT) study, non-White ethnicities showed a higher hazard ratio of 2.14 in males and 1.71 in females. Further, South Asian ethnicities displayed higher hazard ratios (3.0) than white ethnicities to develop T2D (< 2.0) (Rosella et al., 2012).

T2D related health disproportions among ethnicities have also been observed in risk factors such as obesity. Accordingly, recent study suggests a revision of the Body Mass Index (BMI) cut-offs in different ethnic groups enabling better clinical management and further reducing the prevalence of T2D (Caleyachetty et al., 2021). Zhu et al., upon screening 4,906,238 individuals observed that racial/ethnic minorities showed a higher burden of both diabetic and pre-diabetic conditions at lower BMIs than Whites (Zhu et al., 2019). Treatment Options for Type 2 Diabetes in Adolescents and Youth (TODAY) study has observed that non-Hispanic blacks had significantly lower levels of total adiponectin, High Molecular Weight Adiponectin (HMWA) and HMWA-to-total adiponectin ratio at the baseline compared to non-Hispanic Whites and Hispanics (Arslanian et al., 2017). An independent study was conducted to understand the prevalence of coronary heart disease (CHD) in South Asians (Indian, Pakistani and Bangladeshi) settled overseas. The study involved 3,193 men and 561 women residing in London, UK. The findings revealed that the prevalence of T2D was higher in South Asians (19%) when compared with the Europeans (4%). South Asians showed four times greater risk of T2D and 1.5 folds high risk towards the development of CHD than White Europeans. The study also revealed glucose intolerance, hyperinsulinemia, hypertension, low plasma High-Density Lipoproteins (HDL) cholesterol and elevated triglyceride levels in South Asians than Europeans. Independent studies have indicated that insulin resistance is associated with central obesity which is a characteristic feature of South-Asian men and women (McKeigue et al., 1991). Inter-ethnic differences are also documented in T2D associated infections triggered by Herpes Simplex Virus 1 (HSV1), Herpes Simplex Virus 2 (HSV2), Hepatitis A virus (HAV), Hepatitis B Virus (HBV), Hepatitis C Virus (HCV) and *Helicobacter pylori*. In an epidemiological study in The Netherlands, an increased burden of aforesaid infections was observed in subjects of Turkish and Moroccan origin compared with that of Dutch (Hartog et al., 2018). A recent study in British Columbia Hepatitis Testers Cohort indicated increased T2D incidence rates in subjects with HCV infection. Further, East Asians showed a higher impact of HCV associated T2D than South Asians (Jeong et al., 2021).

To address inter-ethnic differences in the manifestation of T2D, several cellular and molecular mechanisms have been hypothesized. South Asians showed the highest risk for developing T2D *via* increased secretion of insulin from pancreatic β-cells whereas African-Americans had higher glucose uptake which was not influenced by insulin (Healy et al., 2015). Hispanic youth with T2D displayed abnormal liver transaminase levels, indicating more risk for developing the non-alcoholic fatty liver disease when compared to African-American and non-Hispanic Whites (Hudson et al., 2012). Integrative gene network analyses of sub-cutaneous adipose tissues revealed significant derangement in the expression of several genes related to mitochondrial energy and metabolism pathways in African-Americans explaining more pronounced insulin resistance than Caucasians (Das et al., 2015). Taken together, accumulating evidence suggests the existence of multiple mechanisms in developing T2D and might be responsible for ethnic differences in developing T2D.

High throughput technologies along with mathematical modelling, machine learning algorithms and functional screening have enhanced our ability to understand the genetic, epigenetic, proteomic and metabolic basis of several diseases including T2D and associated vascular complications. In recent years, metabolomics has evolved as a robust tool to understand the metabolic status of cells/tissues, enabling the discovery of mechanisms, diagnostic biomarkers and therapeutic responses for various diseases including T2D. Ethnicities with distinct genetic backgrounds and lifestyle differentially regulate predisposition, severity and response to T2D. Interdependent effects of genetic interactions with environmental factors leading to poor metabolic health might result in ethnic differences in the prevalence of T2D and associated complications. Ethnicity also contributes to the rate of metabolism and thus, might lead to distinct metabolic signatures during the development and progression of T2D.

**Fig. 1:**
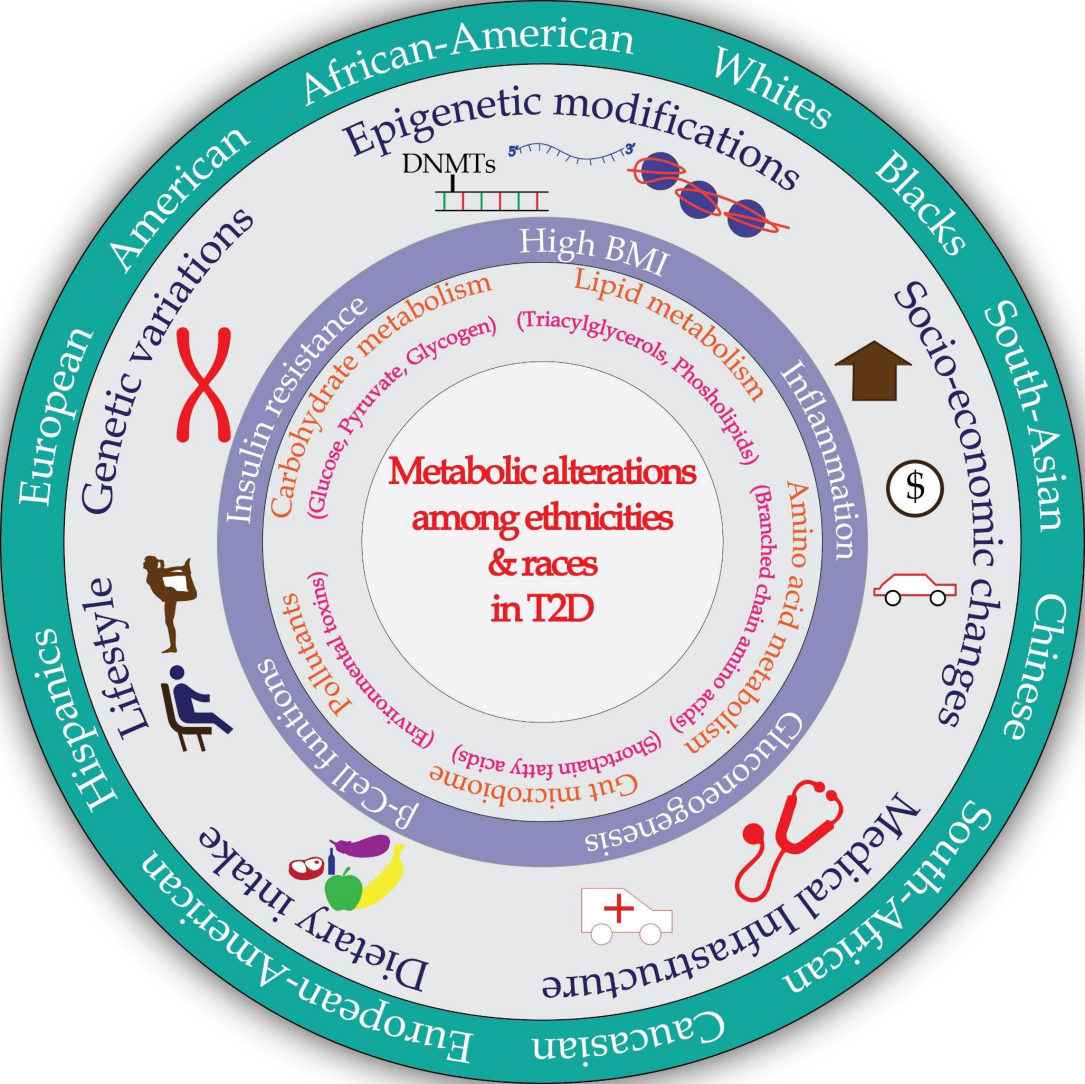
Extrinsic and intrinsic factors contribute to distinct metabolic signatures in T2D among ethnic groups. External elements such as diet and lifestyle modifications influence the metabolome in the T2D. Heterogeneity in the prevalence of T2D across various ethnic groups are also due to socio-economic status and the availability of health infrastructure. Besides, intrinsic factors such as genetic makeup and epigenetic modifications also influence biomolecule metabolism leading to diversity in the metabolite patterns among various ethnic groups in the context of T2D.

Hence, comprehensive and comparative metabolomics analysis between different ethnicities might help to design newer therapeutic strategies unique to a population/ethnicity and further facilitate better clinical management of T2D. In the present review, we explore population-based metabolomics studies in T2D and associated complications with the objectives of (a) comparing metabolic changes among ethnicities; (b) examining how these tissue-specific metabolites/metabolic pathways might contribute to vascular complications, differ between ethnicities and (c) investigating the influence of genetic constitution and dietary patterns on these consequent metabolic changes. Various factors contributing to pathological differences of T2D among ethnicities are summarized in Fig. 1.

## Metabolomics and its applications in understanding disease processes

Metabolomics involves comprehensive analysis of small molecules (< 1.5 kDa) providing both qualitative and quantitative measurements in biological systems (Tan et al., 2016). Besides being potential biomarkers of the disease, metabolites are known to regulate signaling networks or effectors of disease process itself (Johnson et al., 2012). Over the years, multiple approaches have been explored to obtain metabolic profiles of cells/tissues of various organisms to understand biological processes. Mass spectrometry (MS) conjugated either with liquid chromatography (LC) or gas chromatography (GC) and nuclear mass resonance spectroscopy (NMR) are extensively used analytical techniques for metabolomics analysis. Choice of either LC or GC depends on solubility or volatility of the samples and requires further validation of metabolite annotation based on multiple reaction monitoring (Shulaev, 2006). NMR based annotation provides structural insights and further validation is not required and however, experiments require more sample. Hence, due to the wide array of metabolites with their dynamic nature and heterogeneous chemical composition within the cell, a complete analysis requires combination of aforesaid techniques (David & Rostkowski, 2020).

Metabolomics methodologies involve two distinct approaches, untargeted and targeted techniques. Targeted metabolomics involves measurements of defined set of chemically characterized and biochemically annotated metabolites. Using internal standards targeted metabolomics approaches provides exact quantitation of predefined metabolites and hence these techniques helps in understanding enzyme functions, kinetics and end products (Roberts et al., 2012). Untargeted metabolomics protocols provides quantitative and qualitative information of all metabolites in samples including chemical unknowns. Untargeted metabolomics approaches are useful in identifying overall changes in metabolism at a given time and hence, applied in investigating disease processes, finding novel therapeutic targets and drug responses (Joshi et al., 2019), (Patti et al., 2012). Accordingly, over the years, both targeted and untargeted metabolomics approaches have been widely explored in understanding pathophysiology of T2D.

To identify the metabolic diversity across various ethnic groups in the context of T2D, we performed a PubMed search to access the literature. The following are the different keywords were used ‘metabolomics and Type 2 Diabetes’, ‘ethnicities and diabetes’, ‘metabolomics and race’,‘genetic polymorphism and diabetes’, ‘GWAS and diabetes’ and ‘microbiome and diabetes’. Further, based on literature obtained we refined articles only pertaining to Type 2 Diabetes. Subsequently, with respect to T2D and metabolomics related to diverse ethnic groups, we found 26 relevant studies. Out of these 26 studies, 15% of the studies used NMR spectroscopy for their analysis. The remaining 85% of the studies used either GC-MS or LC-MS or a combination of both. Out of these, 45% of studies conducted non-targeted metabolomics and 55% of studies performed targeted metabolomics analysis.

## T2D associated metabolic reprogramming varies among populations

Framingham offspring study demonstrated that euglycemic individuals with elevated levels of Branched-Chain Amino Acids (BCAA) progressed to T2D over 12 years. Further, targeted MS analysis involving participants from a nested case-control study carried out in the identical cohort who progressed towards developing T2D pointed out a significant association between 2-aminoadipic acid and risk for T2D (Razquin et al., 2019). Untargeted metabolome profiling using GC-TOF-MS in the plasma samples of the Gullah-speaking African-American women population revealed that leucine, valine, 2-ketoisocaproate, 2-hydroxybutanoate, histidine, cystine, carbohydrate derivatives and long-chain fatty acids were higher in T2D women when compared with non-diabetic women (Fiehn et al., 2010). The increased levels of HbA1c were positively correlated with elevated levels of leucine and valine. Further, the study revealed that altered metabolism in T2D was due to insulin resistance characterized by reduced efficiency of the Tricarboxylic Acid (TCA) cycle (Fiehn et al., 2010). One of the causes for compromised TCA cycle activity is increased concentrations of 2-hydroxybutanoate which is due to amino acid-derived 2-ketobutanoic acid catalysed by Lactate Dehydrogenase (LDH). The metabolism of biotin is altered due to high 2-hydroxybutanoate. As a consequence of alteration of the BCAA, cysteine catabolism reflecting in TCA cycle disruption was observed (Gall et al., 2010). Metabolic Syndrome in Men (METSIM) study is a population-based study in Finnish men with 4.7 years of follow-up period. The NMR analysis revealed that leucine, alanine, tyrosine, glutamine and isoleucine as predictive markers in assessing the incidence of T2D risk. Further, correlation analysis revealed that isoleucine was strongly associated with insulin sensitivity (Stancáková et al., 2012). Botnia Prospective Study was launched in 1990 with a 10-year follow-up protocol to identify the genetic factors related to the progression of T2D. The participants were residents of the west coast of Finland. A sub-cohort of this population was considered for untargeted and targeted metabolomics analysis. Multivariate analysis suggested a negative association between histidine, glutamine and the (E, E)-isomer of bilirubin with the progression of the T2D in the population (Peddinti et al., 2017). Studies have also reported that bilirubin was negatively correlated with nephropathy in subjects with T2D (Hull & Agarwal, 2014). Elevated levels of glutamate are suggestive of inflammation and oxidative stress and increased glutamate levels also contributes to the deterioration of the pancreatic beta cells in both type 1 diabetes (T1D) and T2D (Davalli et al., 2012).

A meta-analysis of fourteen cohorts comprising 4,592 subjects revealed that leucine (1.89-fold), alanine (1.63-fold) and oleic acid (1.87-fold) were positively correlated to T2D. On the other hand, lysophosphatidylcholines (LPC) 18:0 (20%) and creatinine (37%) showed a negative association with T2D (Park et al., 2018). Metabolomics analysis using UHPLC and GC-MS coupled with a tandem mass spectrometry in a nested case-control study performed among two different cohorts namely, Dongfeng-Tongji (DFTJ) cohort and Jiangsu Non-communicable Disease (JSNCD) cohort showed that alanine, phenylalanine, tyrosine and palmitoyl carnitine as biomarkers of T2D (Qiu et al., 2016). Carnitines, involved in the translocation of fatty acids from cytosol to mitochondria are associated with insulin sensitivity (Mihalik et al., 2010). A targeted metabolomics analysis in Korea Association REsource (KARE) cohort revealed that hexadecanoyl carnitine (C16), glycine, LPC acyl (C18:2) and phosphatidylcholine acyl-alkyl (C36:0) were significantly altered in presence of hyperglycaemia when compared with the control group. Reduced levels of lysoPC (C18:2) are attributed to impaired insulin-stimulated glucose uptake (Lee et al., 2016). Over-nutrition causes excessive fatty acid oxidation and results in mitochondrial stress and also impaired glucose transporter type 4 (GLUT4) signalling (Muoio & Newgard, 2008). KORA (Kooperative gesundheitsforschung in der region Augsburg cohort) of Germany demonstrated that impaired glucose tolerance (IGT) is associated with low levels of glycine and high levels of acetyl carnitine (Wang-Sattler et al., 2012). Qatar Metabolomics Study involving participants of Arabian and Asian descent (QMDiab) performed non-targeted metabolomics analysis in plasma, urinary and salivary samples and revealed that 94 metabolites from all the biofluids are significantly associated with T2D (Yousri et al., 2015).

## Ethnic groups display differences in carbohydrate metabolism in T2D

**Fig. 2:**
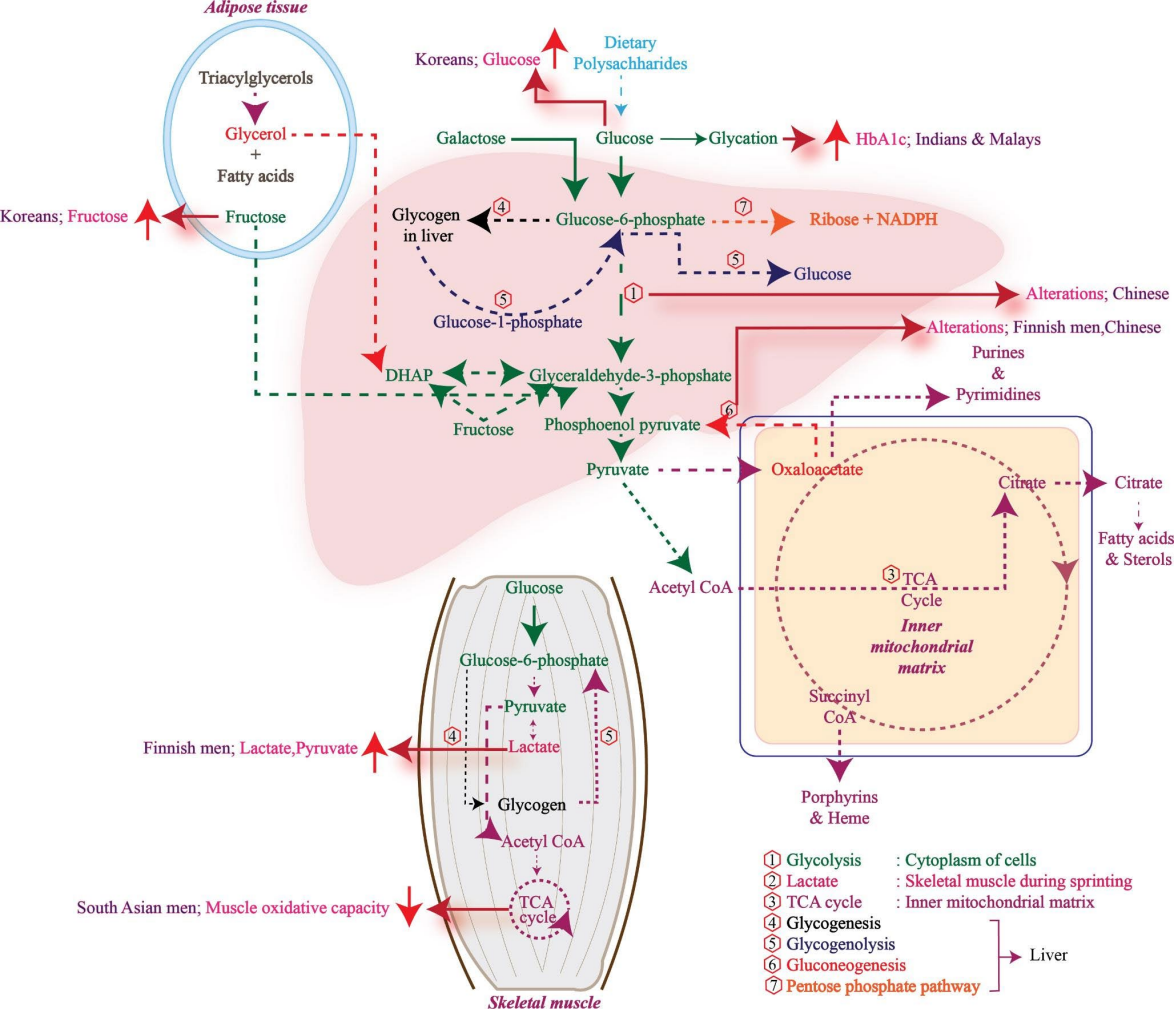
Different ethnicities display distinct alterations in the organ-specific metabolic pathways and their intermediates related to carbohydrate metabolism. In the Chinese population, glycolysis and gluconeogenesis were dysregulated. The fructose and glucose levels were high in Koreans. Glycated hemoglobin was more in Indians and Malays. There was an increase in lactate and pyruvate content in Finnish men. Alterations in TCA cycle metabolism led to decreased muscle oxidative capacity in South Asian men.

Non-targeted metabolomics analysis in the Shanghai Women’s Health Study (SWHS) and the Shanghai Men’s Health Study (SMHS) revealed significant modulation of metabolites associated with pathways regulating glucose homeostasis (glycolysis/gluconeogenesis), BCAA, fatty acids, glycerophospholipids, androgen and bradykinin (Yu et al., 2016). In contrast, the Korean Genome and Epidemiology Study (KoGES) study showed that metabolic indices of valine, alanine, isoleucine, arginine, tyrosine, proline, hexoses and five phosphatidylcholine diacyls were associated with T2D risk which was demonstrated in an 8 year follow up study. Yang et al., using flow injection analysis (FIA)-MS/MS and LC-MS/MS reported a negative correlation between spermine levels and onset of T2D (Yang et al., 2018). Interestingly, spermine serves the role of glycation inhibitor and mediates glucose stimulated insulin release (Welsh & Sjöholm, 1988; Gugliucci & Menini, 2003). A Finnish population based study with a 5-year follow-up showed α1-acid glycoprotein, BCAA and phenylalanine were associated with levels of fasting glycemia. Whereas, alanine, pyruvate, tyrosine and lactate were uniquely associated with post-load glucose. This indicated the altered levels of these precursor molecules might be responsible for insulin sensitivity (Würtz et al., 2013). South Asian ethnic subjects are known to consume a high amount of carbohydrates when compared to Nordic subjects with T2D. Hence, South Asians showed higher production of basal endogenous glucose reflecting increased hepatic insulin resistance (Wium et al., 2013). Taken together, metabolomics analysis in T2D subjects of different ethnicities showed distinct modulations in carbohydrate metabolism and associated intermediates (Fig. 2).

## Branched chain and aromatic amino acids regulate insulin resistance and are differentially modulated among ethnic groups

**Fig. 3:**
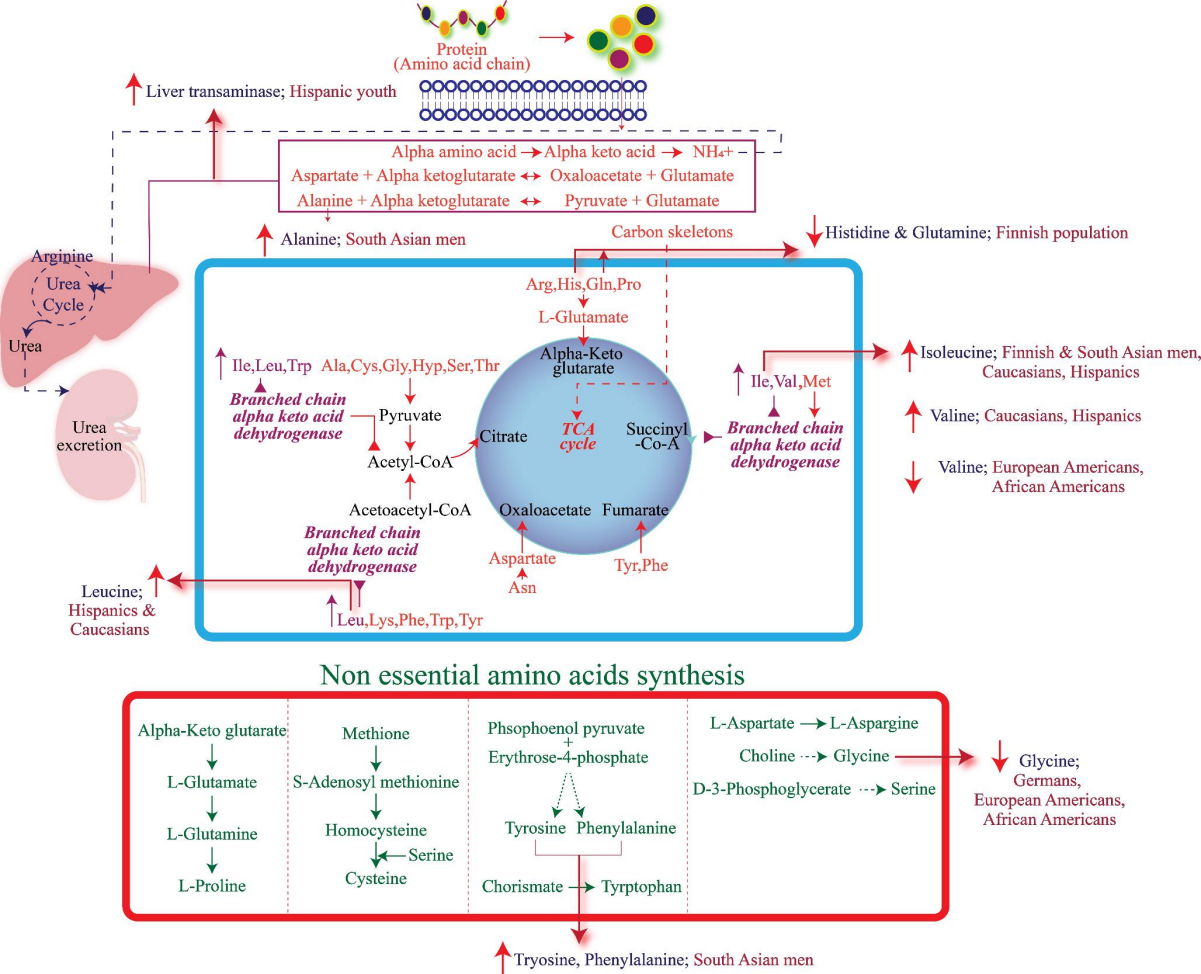
Different ethnic groups display distinct alterations in the amino acid metabolism and their intermediates. Lower levels of histidine, glutamine and higher levels of isoleucine were observed in the Finnish population. Valine and glycine levels were low in European Americans and African-Americans. Increased levels of liver transaminases were observed in Hispanic youth. Alanine, isoleucine, tyrosine, phenylalanine were elevated in South Asian men. Hispanics and Caucasians had higher amounts of leucine, valine and isoleucine.

T2D is associated with significant alterations in amino acid metabolism. The SABRE (Southall And Brent REvisited study) aimed to understand metabolic status in T2D among various ethnic groups. The participants were Europeans (n = 1,279) and South Asians (n = 1,007) (20% of Hindus, 15% of Muslims, 52% of Punjabi Sikhs, and 13% of other South Asians). The study demonstrated the relationship between circulating amino acids and the onset of T2D. Predisposing factors of T2D such as alcohol consumption and smoking were less in the South Asian ethnic groups. Isoleucine, tyrosine, alanine and phenylalanine levels were significantly elevated in the South Asian cohort. However, these amino acids showed cross-sectional correlations with glycemic index and insulin resistance in both ethnic groups. After a 19 year follow-up, the metabolite profile showed a strong association of tyrosine with the incidence of T2D in South Asians (Tillin et al., 2015). The association of increased BCAAs is attributed to dysregulation of amino acid metabolism in the liver, kidneys, muscles, or adipose tissues (Krebs et al., 2002; Tremblay et al., 2007). When compared with the Europeans, South Asians reportedly have low muscle mass, more hepatic fat and high central obesity (Chowdhury et al., 1996; Petersen et al., 2006). Studies have shown that, when compared with Caucasians, adipocytes are relatively large in South Asian men. The levels of plasma adiponectin in South Asian men were inversely correlated with that of the adipocyte size. The study concluded that insulin resistance in South Asian men may be due to the hypertrophy of the adipocytes and truncal adiposity rather than visceral fat (Chandalia et al., 2007). A sub-cohort of METSIM study in Finland, also showed that ketone bodies were associated with abnormal glucose tolerance. In a prospective 13-year follow-up study, metabolite profiling was performed in South African women segregated into normo-glycemic, T2D and impaired fasting glycaemic groups. The study indicated that metabolic products of phospholipids and bile acids along with BCAA’s were significantly high in subjects who had developed T2D after 13 years. The levels of these metabolites were unaltered in subjects who did not progress towards T2D. During the follow-up, it was observed that at the onset of T2D participants had higher levels of LPC(C18:2) levels (Zenget al., 2019). Interestingly, LPCs are involved in glucose-dependent insulin secretion by the activation of G-protein coupled receptors (GPR) (Soga et al., 2005). It is also evident that compared to White South African women, higher levels of insulin are secreted by pancreatic beta-cell in black South African women (Goedecke et al., 2009). Deoxycholic acid and glycodeoxycholic acid levels were lower at baseline in subjects who progressed towards T2D during the follow-up. Bile acids are known to enhance the absorption of fats. Failure to reabsorb the bile acids results in altered signalling of glucagon-like peptide-1 and insulin (Brighton et al., 2015). Hence, Deoxycholic acid and glycodeoxycholic acid might serve as predictive markers of T2D (Zeng et al., 2019). The Insulin Resistance Atherosclerosis Study (IRAS) study is one of the largest comprehensive epidemiologic studies that address insulin resistance and atherosclerosis which are the cause and consequence of T2D respectively (Wagenknecht et al., 1995). Targeted profiling of BCAA as a part of IRAS in a multi-ethnic population involving African-Americans, Hispanics and Caucasians and performing multivariable-adjusted odds ratio per 1-SD increase in plasma BCAA, revealed that insulin sensitivity had a higher association with BCAA. The association was stronger in Caucasians and Hispanics when compared with the participants in other two ethnicities (Lee et al., 2016). A Multi-ethnic study was conducted using targeted metabolomics in European American, Hispanic, and African-American subjects. Both the extremes of insulin resistance values were observed among different ethnicities. Subjects with insulin resistance showed decreased levels of glycine and higher concentrations of valine, leucine, phenylalanine, glutamate and glutamine. These levels were more pronounced in European Americans and African Americans but not in Hispanics (Palmer et al., 2015). The altered products of amino acid metabolism among different ethnic groups are summarized in Fig. 3.

## Lipids regulating immuno-metabolic axis in T2D varies among ethnicities

**Fig. 4:**
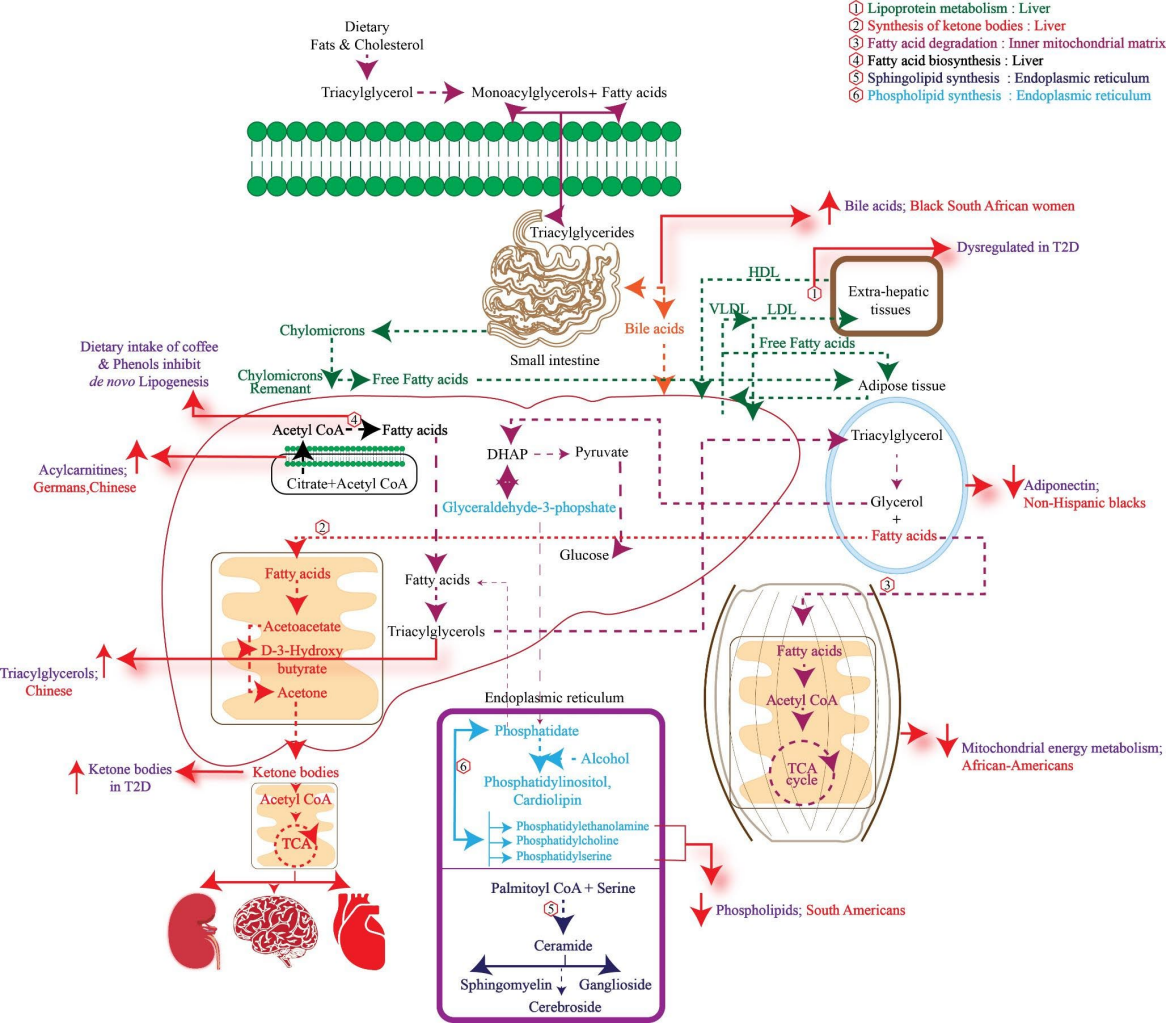
The fate of dietary fats and cholesterol along with biogenesis and degradation of fatty acids are represented. The ethnicity-specific metabolites of lipid metabolism with their site of synthesis/breakdown are highlighted. Metabolic synchronization between the liver, skeletal muscle, adipose tissue and intestine are derailed during the T2D. Black South African Women had increased levels of bile acids. Acylcarnitines and triacylglycerols were high in Chinese. The levels of phospholipids were low in South Americans. Dysregulated mitochondrial energy metabolism was observed in African-Americans. Non-Hispanic blacks had low levels of adiponectin.

One of the pathological changes associated with T2D is persistent low-grade inflammation associated with increased levels of pro-inflammatory mediators (Ellulu et al., 2017). Inflammation is one of the important factors triggering insulin resistance in various tissues (Balakrishnan et al., 2018). Accordingly, efforts are made to treat chronic inflammation and restore immune homeostasis by promoting ‘resolution of inflammation in diabetic conditions (Brennan et al., 2018). Accumulating evidence indicates that lipoprotein metabolism is dysregulated in T2D which may lead to alterations in the peripheral circulation of Polyunsaturated Fatty Acids (PUFA) (Boden & Laakso, 2004). A nested case-cohort study within the participants of the PREDIMED trial (Prevention with Mediterranean Diet) which involved populations from different geographies of South America such as South (Andalusia and Canary Islands), North (Navarra and Basque country), East (Valencia and Balearic Islands), North-East (Catalonia). The Targeted lipidomic approach showed increased levels of cholesterol esters, LPCs, Sphingomyelins (SMs), Phosphatidylcholine-Plasmalogens (PC-PLs), and lysophosphatidylethanolamines were negatively associated with the risk of T2D (Razquin et al., 2018). This is because LPCs are known to reduce blood glucose levels and have anti-inflammatory effects (Lehmann et al., 2013). Plasmalogens possess antioxidant, anti-apoptotic and anti-inflammatory properties (Huynh et al., 2017). SMs play a vital role in mitochondrial function and efficient glucose-stimulated insulin secretion which was confirmed in SM synthase 1 knockout mouse models (Yano et al., 2011). A multi-cohort nested case-control study involving European Prospective Investigation into Cancer and Nutrition (EPIC)-InterAct study showed that high concentrations of linoleic acid, odd chain fatty acids, and Very-Long-Chain Fatty Acids (VLSFAs) are associated with a lower occurrence of T2D. This in turn is mediated by genetic and epigenetic factors. Along with this higher concentration of endogenously synthesized fatty acids such as 18:2n-6, odd-chain Saturated Fatty Acids (SFAs) and VLSFAs and decreased concentrations of long-chain SFAs and Mono Saturated Fatty Acids (MUFAs) reduced the risk of T2D (Imamura et al., 2017). A multi-cohort study in Germany with the cohorts labelled, a) EPIC Potsdam study is an integral part of multicentre EPIC study b) Cooperative Health Research in the Region of Augsburg study c) Tübingen Family study for T2D involved analysing oral glucose tolerance and targeted serum metabolomics. Analysis showed that the elevated levels of diacyl-phosphatidylcholines C36:1, C32:1, C40:5, and C38:3 and decreased concentrations SM C16:1, acyl-alkyl-phosphatidylcholines C42:5, C40:6, C44:4, C44:5 and C34:3 and LPC C18:2 were associated with T2D (Floegel et al., 2013). Phospholipids are an integral part of the cell membrane and are involved in several cellular processes. Choline is the primary precursor for the production of diacyl-phosphatidylcholines and acyl-alkyl-phosphatidylcholines which are involved in the production of Very Low Density Lipoprotein (VLDL), HDL and are associated with the antioxidant activity (Wallner & Schmitz, 2011; Cole et al., 2012). Studies in mouse models showed dietary intake of choline was necessary for improved glucose tolerance (Raubenheimer et al., 2006). The METSIM study revealed that serum levels of Free Fatty Acids (FFAs), glycerol, SFAs, and MUFAs and n-7, n-9 FAs are associated with T2D (Mahendran et al., 2013). SFAs are further desaturated into MUFAs and hence correlated with T2D in the population consuming the Western diet (Sundström et al., 2001; Kouki et al., 2011). The intake of SFAs is also related to insulin resistance (Lichtenstein & Schwab, 2000). Targeted serum lipidomics in a Chinese cohort with T2D revealed an association between triacylglycerols, PUFA’s and cholesteryl esters with T2D (Lu et al., 2019). A six-year follow-up study in the Chinese population with T2D without any previous history of cardiovascular diseases showed an alteration in the metabolic profile in T2D subjects. Untargeted metabolomics showed that BCAA such as leucine, isoleucine, valine and non-esterified fatty acids such as palmitic acid, oleic acid, linolenic acid and stearic acid were significantly associated with T2D. It was also demonstrated that metabolites such as amino malonic acid, urea, proline, 3-carboxy-4-methyl-5-propyl-2-furanpropionic acid, LPI (16:1) and glycerol are considered in predicting the risk of T2D (Lu et al., 2016). A population-based targeted metabolomics study in Beijing and Shanghai revealed that acylcarnitines, a component of the energy production unit were significantly associated with T2D (Sun et al., 2016). Afro-Caribbean subjects with T2D had a lower risk of myocardial infarction in comparison with Whites and South Asians. Gray et al., observed that delta-6 desaturase (D6D) activity was significantly low in Asian Indian women in comparison with White Europeans (Gray et al., 2013). SFAs are converted to monounsaturated equivalents by the action of the lipid desaturase enzyme. Delta-6 and delta-5 desaturase (D5D) are important for desaturations (Gray et al., 2013). An untargeted metabolomics study in the Indian populations residing in North and South Dakota, Oklahoma and Arizona reported that 2-hydroxybiphenyl was associated with risk of T2D. However, 2-hydroxybiphenyl has large-scale industrial applications and is an environmental toxin indicating the role of pollutants in developing T2D (Zhao et al., 2015). Ethnicity specific alterations in lipid metabolism are summarized in Fig. 4.

## Inter-ethnic genetic variations in genes regulating amino acid and lipid metabolism might contribute to differential metabolic reprogramming

Over the years, Genome Wide Association Studies (GWAS) have indicated ethnicity based genetic markers associated with a variety of diseases including T2D (Ali, 2013). A meta-analysis of 26,676 cases of T2D and 132,532 controls belonging to European ancestry revealed 13 novel loci (*p* < 5 × 10^− 8^) associated with T2D. These loci are associated with gastric inhibitory peptide (*GIP*), major histocompatibility complex, class II, DQ alpha 1 (*HLA-DQA1*), neurexin 3 (*NRXN3*), C-Maf Inducing Protein (*CMIP*) and glucagon Like Peptide 2 Receptor (*GLP2R*) genes (Scott et al., 2017). One of the significant associations was observed at rs1182436 which is upstream of the motor neuron and pancreas homeobox 1 (*MNX1*) gene which plays a role in pancreatic hypoplasia and neonatal diabetes (Flanagan et al., 2014). A missense variant (rs17681684) close to the *GLP2R* gene has been shown associated with glucose tolerance (Guan, 2014). A study conducted on Japanese individuals has revealed that cyclin dependent kinase inhibitor 2B (*CDKN2B*) (rs10811661), CDK5 regulatory associated protein 1-like 1 (*CDKAL1*) (rs7756992), solute carrier family 30 member 8 (*SLC30A8*) (rs1326663), insulin like growth factor 2 mRNA binding protein 2 (*IGF2BP2*) (rs4402960), C2 calcium dependent domain containing 4 A (*C2CD4A/B*) (rs7172432), potassium voltage-gated channel subfamily Q member 1 (*KCNQ1*) (rs2237892), insulin receptor substrate 1 (*IRS-1*) (rs2943641), glucokinase regulator (*GCKR*) (rs780094) and potassium inwardly rectifying channel subfamily J member 11 (*KCNJ11*) (rs5219) were associated with predisposition to T2D. Further, Iwata et al., demonstrated a significant association between genetic risk score (P = 5.9 × 10^− 21^) and early onset of T2D (Iwata et al., 2012). A population based study conducted in India has shown that rs998451 (odds ratio (OR) 1.56; P = 6.3 × 10^− 12^) at 2q21 locus which is close to transmembrane protein 163 (*TMEM163*) is associated with reduced levels of fasting plasma insulin (Tabassum et al., 2013). Zinc transporter protein member 8 (ZnT-8) (*SLC30A8*) belonging to the zinc transporter family is involved in insulin signalling. A meta-analysis was conducted to find the association between *SLC30A8* polymorphism (rs13266634) and the risk of T2D. The study demonstrated that Europeans (OR = 1.15, 95% CI (Confidence Interval) 1.11–1.18, p < 0.001) and Asians (OR = 1.15, 95% CI 1.11–1.19, p < 0.001) harbouring this single nucleotide polymorphism (SNPs) are at increased risk of developing T2D (Jing et al., 2011). Ectonucleotide pyrophosphatase/phosphodiesterase 1 *ENPP1/PC1* variants have been shown associated with insulin resistance leading to increased risk for T2D. Genotyping analysis revealed that *ENPP1/PC1 K121Q* variant was relatively higher in African-Americans (78.5%) and Hispanics (21.9%) in contrast to the non-Hispanic White group. The study also stated that African-Americans (14.1%) and Hispanics (11.7%) showed a high prevalence of T2D when compared with non-Hispanic Whites (Chandalia, et al., 2007).

KORA S4 survey which was conducted in Germany identified several genetic variants linked with altered metabolite levels. A total of 163 metabolic traits were measured in the population. The variants of electron transfer flavoprotein dehydrogenase (*ETFDH*), monocarboxylate transporter 9 (*SLC16A9*), correspond to genes encoding solute carriers. *ACADS, ACADM and ACADL* genes are associated with beta (β)-oxidation of fatty acids. Fatty acid desaturase 1 (*FADS1*), and elongation of very-long-chain fatty acids-like 2 (*ELOVL2*) are linked with the biogenesis of polyunsaturated fatty acids and serine palmitoyl transferase long chain base subunit 3 (*SPTLC3*) is related to phospholipid biosynthesis (Illig et al., 2010). The majority of these loci were validated which were closer to the genes regulating enzymes of β-oxidation, amino acid metabolism, fatty acid and phospholipid biosynthesis. The study indicated a 36% variance in the metabolite levels contributed by genetic variations (Gonzalez-Franquesa et al., 2016). Peroxisome proliferator-activated receptor gamma (*PPARG)* alters the rate-limiting step of fatty acid biosynthesis mediated by stearoyl-CoA desaturase (Brown et al., 2008; Wahli & Michalik, 2012) and also regulates carbohydrate metabolism (Radha & Mohan, 2007). A meta-analysis revealed that the minor allele of the rs1801282 (Pro12Ala) variant of *PPARG* decreases the risk of T2D. This protective effect is significantly observed in Europeans. The OR and CI for homozygous (OR: 0.74, 95% CI: 0.59–0.92), for heterozygous (OR: 0.88, 95% CI: 0.79–0.98), under allelic are (OR: 0.82, 95% CI: 0.73–0.91), for co-dominant (OR: 0.88, 95% CI: 0.82–0.95), for recessive (OR: 0.75, 95% CI: 0.61–0.93), for additive (OR: 0.76, 95% CI: 0.58–0.98) and for dominant (OR: 0.86, 95% CI: 0.77–0.96). East Asians had an co-dominant (OR: 0.80, 95% CI: 0.65–0.98) and under allelic (OR: 0.80, 95% CI: 0.63–1.00) variant (Sarhangi et al., 2020). Several case-control studies revealed that Pro12Ala variant of *PPARG* in East Asia (Japanese) (Mori et al., 2001), Greater Middle Eastern (Motavallian et al., 2013) and in the populations with European lineages such as Czech (Pintérová et al., 2004), Scottish (Doney et al., 2004) and Finnish (Douglas et al., 2001) reduces the risk for developing T2D. Pima Indians are known to have the highest prevalence of T2D (Hanson et al., 1998). A population specific study was conducted involving 332 nuclear families of Pima Indians. The study screened genetic variations associated with phospholipase A2, Group IVA (*PLA2G4A)* which falls in the category of calcium-dependent phospholipases (cPLA2s) and are involved in the cleaving of arachidonic acid from phospholipid membranes. Wolford et al., identified a C to G variant associated with *PLA2G4A* in the Pima Indians, which leads to the substitution of phenylalanine with leucine (Wolford et al., 2003). This enzymatic activity of cPLA2s helps to provide arachidonic acid as a precursor molecule for the biogenesis of eicosanoids (Clark et al., 1991). The intermediates and the compounds produced in this pathway play a vital role in insulin secretion which is influenced by secretagogues (Parker et al., 1996; Ahrén et al., 2000). Study participants with C to G variant had lesser endogenous glucose output and low mean basal glucose oxidation. Under normal physiological conditions, the variant genotype containing individuals had higher levels of lipid oxidation which reflects the effect of SNP in the *PLA2G4A* gene (Wolford et al., 2003).

The meta-analysis involving two cohorts (a) Asian Genetic Epidemiology Network (AGEN) and (b) Diabetes Meta-analysis of Trans-ethnic Association Studies (DIAMANTE) with 77,418 T2D subjects and 356,122 controls revealed 301 distinct signals associated with 183 loci in T2D subjects. When gender based stratification was performed aldehyde dehydrogenase (*ALDH2*) (rs12231737) was associated with males (P_males_=5.8 × 10 − 27). One of the significant missense variants associated with rs12231737 (P_het_=2.6 × 10 − 19) is rs671 (*ALDH2* Glu504Lys: OR = 1.17, risk allele frequencies (RAF) = 77.7%, 95% CI 1.14–1.20, P_males_=1.5 × 10 − 24). This variant is known to reduce *ALDH2* activity (Spracklen et al., 2020). *ALDH2* is an aldehyde dehydrogenase 2 family member, which catalyses the conversion of acetaldehyde into acetic acid. *ALDH2* 504Lys allele is associated with high BMI, increased tolerance of alcohol, BP, HDL, and decreased low Density lipoprotein (LDL) and cardiovascular risk in East Asians (Xu et al., 2010; Takeuchi et al., 2011). A population-based study was performed to understand the loss-of-function variants associated with the adenylyl cyclase 3 (*ADCY3*) gene. The study participants were a part of two different cohorts; the B99 cohort and Inuit Health in Transition (IHIT) located in Greenland. One of the variants was known to destroy a splice-acceptor site in exon 14 of *ADCY3* encoding adenylate cyclase. This catalyses the formation of cyclic adenosine monophosphate (cAMP) from adenosine triphosphate (ATP). The study showed that loss-of-function variants in Greenlandic homozygous (*ADCY3* c.2433-1G > A) resulting in a phenotype characterized by truncal adiposity and metabolic alterations associated with insulin resistance, dyslipidemia and T2D (Grarup et al., 2018). To identify the influence of genetic variations on the health of U.S. military veterans, a Million Veteran Program (MVP) was launched in 2011. The study consisted of non-Hispanic Blacks, Hispanics and non-Hispanic Whites (Klarin et al., 2018). Based on GWAS and subsequent Transcriptome wide association studies (TWAS) Gandotra et al., identified the presence of rare missense mutation in the gene coding for Perilipin-1 (*PLIN1* p.Leu90Pro). Subjects with these variants showed higher levels of plasma HDL. Perilipin-1 in humans is vital for the formation of lipid droplets, triglyceride storage and metabolism of FFAs (Gandotra et al., 2011). MVP study also pointed out that brain-derived neurotrophic factor (*BDNF*) downstream variants were linked with triglycerides and HDL levels and imply the association of this gene with T2D and metabolic syndrome (Rani et al., 2017). Taken together, these genetic variations demonstrate inter-ethnic differences leading to dyslipidaemia (Klarin et al., 2018). Serum adiponectin level is one of the factors which inversely correlates with blood glucose, indices of insulin resistance and T2D (Hivert et al., 2008). Polymorphism in the adiponectin gene (*ADIPOQ*) is correlated with serum levels of adiponectin in most of the studies (Manning, et al., 2008; Ling et al., 2009; Menzaghi et al., 2007). Studies conducted on East Asians have revealed that cadherin 13 (*CDH13*) is linked with adiponectin levels (Jee et al., 2010). A meta-analysis combining participants from three ethnic backgrounds such as White Europeans (n = 29,347), East Asians (n = 1,776) and African--Americans (n = 4,232) has revealed that common variants of *CDH13* and *ADIPOQ* loci regulate the adiponectin levels. A variant of *ADIPOQ*; rs6810075 [T] is associated with the population of European descent. These variations, in turn, alter insulin, triglycerides, post-prandial glucose, waist-to-hip ratio and HDL in the individuals. Genome wide significance of *ADIPOQ* locus was observed in African-Americans whereas in East Asians significance was observed at *ADIPOQ* and *CDH13* loci (Dastani et al., 2012). Interestingly, *CDH13* overexpression in vascular endothelium led to reduced responsiveness to insulin to activate endothelial nitric oxide synthase (eNOS) (Philippova et al., 2010). GPR induced protein that interacts with MAP kinases is encoded by *TRIB1*. MAP kinases are involved in regulating chemotaxis and proliferation of vascular smooth muscle cells (Kiss-Toth et al., 2004). Atherosclerosis is a macro-vascular complication associated with T2D. The expression of *TRIB1* is elevated during atherosclerosis vascular smooth muscle cells (Sung et al., 2007). A GWAS was performed on the United States of America (USA) based Hispanic/Latino background groups to understand the genetic underpinning of circulating metabolites. The study revealed 46 different loci regulating the metabolome. One of the variants in dedicator of cytokinesis 7 (*DOCK7*) gene rs10889335, which is linked with phosphatidylinositol 1-stearoyl-2-arachidonoyl-GPI (18:0/20:3). *DOCK7* is co-localized with low levels of angiopoietin-like protein 3 (*ANGPTL3*) in the liver. *ANGPTL3* is associated with lipid metabolism and is known to cause type 2 hypobetalipoproteinaemia. Variants of *TRIB1* (rs2954029, rs2954021, rs17321515) are associated with LDL, CHD and HDL in Asian and European populations (Willer et al., 2008; Chasman et al., 2009; Teslovich et al., 2010; Waterworth et al., 2010; Park et al., 2011). Hispanics, an ethnic group are more prone to cardiometabolic diseases (Feofanova et al., 2020). *FADS1* and its corresponding variant rs174554 are associated with elevated levels of 1-palmitoyl-2-stearoyl-GPC (16:0/18:0) which belongs to phosphatidylcholine species. These lipid molecules play a vital role in insulin resistance (Chang et al., 2019). HbA1c is a widely accepted marker for diagnosis and glycaemic control in T2D. GWAS performed as a part of the Singapore Malay Eye Study and Living Biobank study indicated deletions at *SLC4A1* (rs769664228) are associated with reduced HbA1c levels. This variant has been significantly associated with the Malay population (Chai et al., 2020). Metabolic syndrome is one of the risk factors for T2D. Analysis of serum metabolome in a subset of the American population, which included Whites, Blacks and participants of unknown race revealed that BCAA was significantly associated with T2D. Interestingly, these findings are supported by a study that shows that a genetic variant of the (protein phosphatase, Mg^2+^/Mn^2+^ dependent 1 K) (*PPM1K*) gene which activates Branched Chain Amino Acid ketoacid dehydrogenase (BCKD) is associated with T2D (Lotta et al., 2016). Independent studies have demonstrated decreased expression of branched-chain aminotransferase and BCKD causes elevated levels of BCAAs (She et al., 2007).

## Does distinct dietary and microbiome patterns reflect on metabolome resulting in ethnic disparities in T2D?

Diet significantly influences genetic and epigenetic regulation of gene expression and thus, an imbalanced diet considerably contributes to the pathogenesis of metabolic disorders including T2D. Diet consisting of high sodium content, high meat and trans fats have been demonstrated as a high-risk factor for T2D and obesity (Murray et al., 2013). The NHS which tracked 84,941 female nurses between 1980 and 1996 reported that poor diet, alcohol intake and smoking are significantly associated with a high risk for T2D. A diet high in polyunsaturated fat, cereal fibre and low in glycaemic load and trans-fat proved to maintain good euglycemic levels (Hu et al., 2001). A meta-analysis of 15,043 T2D of 310,819 participants revealed that sugar-sweetened beverages with 1–2 servings per day increased the risk of T2D by 26% (Malik et al., 2010). A prudent diet with an appropriate amount of calorie intake and rich in n-3 fatty acids instead of saturated fats and avoiding intake of tobacco and alcohol helped to prevent diabetes and associated vascular complications in Indians (Singh et al., 1997). Dalda, a variety of vegetable ghee, is known to contain high trans fats and is widely consumed in India along with other South Asian geographies (Popkin, 2001). Intake of the higher amount of trans fats is linked with insulin resistance and elevation of several inflammatory mediators which further leads to endothelial dysfunction and may result in vascular complications (Lopez-Garcia et al., 2005). The SWHS involving 64,227 Chinese women without a history of T2D revealed that intake of rice and high glycemic foods may increase the probability of developing T2D in women (Villegas et al., 2007). Meta-analyses and systematic reviews from the Nutrition and Chronic Diseases Expert Group (NutriCoDE) identified trans-fats, sugar-sweetened beverages and processed meats are widely responsible for cardiovascular outcomes. In contrast, intake of a prudent diet showed protective effects (Micha et al., 2017). Analysis of the data from the cross-sectional study of the Asian Indians a part of the Metabolic Syndrome and Atherosclerosis in South Asians Living in America (MASALA), a pilot study in the USA revealed that western/non-vegetarian diet was associated with higher levels of BCAA, aromatic amino acids and short-chain acylcarnitines. Higher levels of these metabolites are related to elevated levels of fasting glucose and lower levels of total adiponectin. This study revealed that diet showed a major impact on the development of cardiovascular anomalies (Mozaffarian, 2016). A parallel double blinded study demonstrated intake of sucrose-sweetened beverages and high-fructose corn syrup (3 servings/day for 16 days) significantly increased the hepatic lipid content, plasma lipids concentrations, uric acid and lipoproteins. A significant reduction in Matsuda ISI index along with decreased insulin sensitivity was observed in the subjects who consumed these beverages (Sigala et al., 2021).

A meta-analysis involving 16 randomized control trials has shown that physical activity, reduced caloric intake and dietary education showed beneficial outcomes on cardiovascular complications associated with T2D. Results of the Look AHEAD (Action for Health in Diabetes) trial have revealed that decreased caloric intake reduced the levels of HbA1c further minimized cardiovascular risk factors (Wing et al., 2013). Da Qing Diabetes Prevention Study which involved a six-year lifestyle intervention and a follow-up for 23 years in Chinese adults with IGT revealed that mortality associated with cardiovascular diseases was reduced by 41% (Li et al., 2014). A comprehensive meta-analysis revealed that intake of whole grain was associated with lowering T2D to an extent of 21% (de Munter et al., 2007). The results of the INTERHEART study revealed that diabetic individuals upon dietary intake of fruits and vegetables accounted for reduced myocardial infarction in the study participants belonging to Asia, Europe, the Middle East, Africa, Australia, North America and South America (Yusuf et al., 2004). A comprehensive meta-analysis summarized the beneficial effects of different dietary patterns on cardiometabolic outcomes was conducted by The Diabetes and Nutrition Study Group (DNSG) of the European Association for the Study of Diabetes (EASD) (Kahleova et al., 2019). The Mediterranean diet (Becerra-Tomás et al., 2020), Dietary Approaches to Stop Hypertension (DASH) diet (Chiavaroli et al., 2019), Portfolio diet (Chiavaroli et al., 2018), Nordic diet (Ramezani-Jolfaie et al., 2019) and Vegetarian diet (Glenn et al., 2019) is known to reduce the cardio metabolic outcomes. These prudent diets are known to reduce the levels of both HbA1c and LDL.

Studies in diabetic individuals such as the PREDIMED trial which involved the intake of a Mediterranean diet prevented cardiovascular anomalies by 31% (Estruch et al., 2013). A randomized double-blinded crossover study was conducted in European and South-Asian descents to understand the influence of L-arginine supplementation for six weeks on skeletal muscle and brown adipose tissue metabolism. The findings indicated improved glucose tolerance in European men whereas South Asian men did not respond to the supplementation. Further, *Boon et al.*, demonstrated that South Asian men possessed lower skeletal muscle oxidative capacity compared to European men (Boon et al., 2019). L-Arginine is the precursor for the formation of nitric oxide mediated by eNOS. Interestingly, an independent study showed that endothelial cells isolated from South Asian men displayed lower expression of eNOS than men of European ethnic groups (Cubbon et al., 2014). Histidine-influenced abrogation of hepatic glucose production is a widely used treatment for T2D (Kimura et al., 2013). Supplementation of the glutamine was protective in the context of T2D (Mansour et al., 2015). Specialized pro-resolving lipid mediators (SPMs), derived from dietary PUFA facilitated the restoration of immune homeostasis. Peripheral circulation of vital PUFA such as linoleic acid and arachidonic acid is carried out by lipoproteins and the content of PUFAs varies among lipoproteins (Ander et al., 2003). SPMs belonging to the family of ‘protectins’, ‘lipoxins’, ‘resolvins’ and ‘maresins’ have recently been demonstrated for their active role in adaptive immune response (Duffney et al., 2018). SPMs reduce excessive leukocyte infiltration and enhance the resolution of immune response by limiting the entry of pro-inflammatory signalling molecules (Recchiuti et al., 2019). SPMs are known to be protective against diabetic complications such as atherosclerosis, wound healing, nephropathy and retinopathy. Also, the activity of enzymes involved in the formation of SPMs were altered in T2D (Russo et al., 2013). Dietary intake is known to mediate fatty acid metabolism. Intake of coffee and polyphenols inhibit *de novo* lipogenesis (Murase et al., 2011).

T2D is associated with significant dysbiosis of the gut microbiome. Trimethylamine-N-oxide (TMAO) is a gut metabolite produced as a result of intestinal microbial metabolism (Roy et al., 2020). Trimethylamine is typically produced by gut bacteria due to the metabolism of choline, L-carnitine and phosphatidylcholine in the intestine. The trimethylamine is further converted into TMAO in the liver (Roy et al., 2020). Several independent studies have shown the association of T2D with elevated levels of TMAO (Lever et al., 2014; Tang et al., 2014; Dambrova et al., 2016; Shan et al., 2017). A longitudinal cohort study entitled, The Oral Infections, Glucose Intolerance and Insulin Resistance Study (ORIGINS) was conducted to assess the link between TMAO and T2D. The study recruited 300 subjects of both genders belonging to non-Hispanic Black, non-Hispanic White, Hispanic and other ethnic groups. The participants were free of T2D and aged between 20 and 55 years at the baseline. The association between the TMAO and the biomarkers of the T2D were analysed after 2 years of recruitment. The results demonstrated that TMAO levels were moderately associated with Hispanics when compared with the rest of the ethnic groups. The statistical analysis revealed that TMAO levels are significantly associated with the prevalence of prediabetes (Roy et al., 2020). T2D is also a risk factor for heart failure (Dunlay et al., 2019). The UK Leicester cohort study recruited Caucasians (n = 842, 77%), South Asians (n = 129, 12%) and Japanese cohort (n = 116, 11%) to understand the role of ethnicity in the context of elevated levels of TMAO and heart failure. The study revealed that elevated TMAO levels resulted in heart failure in Caucasian subjects in comparison with the other ethnic groups (Yazaki et al., 2020).

One of the by-products of carbohydrate metabolism influenced by the gut microbiome is the synthesis of Short chain fatty acid (SFAs) such as propanoic acid, butyric acid and *inter alia*. These SFAs are known to protect the intestinal epithelium (Saad et al., 2016). SFAs are synthesized *by Bacteroides, Clostridium, Bifidobacterium, Eubacterium, Streptococcus* and *Peptostreptococcus* (Ma et al., 2019). SFAs metabolism alterations are associated with increased expression of NF-κB and production of inflammatory mediators such as interleukin-8 (IL-8) and TNF-α by neutrophils and macrophages (Tan et al., 2014). The reduced production of SFAs inhibited the differentiation of T-cells into T-regulatory cells which subsequently resulted in inhibition of IL-10 leading to the inflammatory response (Lopez et al., 2014). Reduced SFAs also led to increased phosphorylation of tyrosine and serine kinases that resulted in activation of the interferon- γ (IFN-γ)/STAT1 signalling pathway contributing to intestinal inflammation (Klampfer et al., 2003). A Metagenome-Wide Association Study (MGWAS) based on deep shotgun sequencing was conducted to assess microbial dysbiosis in the context of T2D. The study participants were Chinese individuals and analysis revealed lower levels of butyrate producing bacteria in the T2D subjects when compared with the controls (Qin et al., 2012). The Asian Indian phenotype of T2D is different from that of European descent. This is because of a difference in body fat, inflammatory markers and lipid profile (Gujral et al., 2013; Unnikrishnan et al., 2014). This geography-specific phenotype is linked with the unique dietary intake of the Indian’s which is in turn reflected in the profile of the gut microbiome (Bhute et al., 2016; Das et al., 2018; Kalyana Chakravarthy et al., 2018; Tandon et al., 2018). A comparison amongst healthy individuals and T2D subjects revealed that *Escherichia* is abundantly present in subjects with T2D in the Indian population (Bhute et al., 2017; Pushpanathan et al., 2016). A trans-ethnic study involving pre-diabetic subjects from Denmark (White European ethnicity) and India has reported that *Megasphaera* is one of the species which is very abundant in IGT subjects in India. The study showed more alpha diversity in the Danish cohort. (Nishikawa et al., 2009; Guinane & Cotter, 2013). The host-gut microbial axis is influenced by lifestyle (Rothschild et al., 2018), dietary intake (David et al., 2014), medications (Blaser, 2016). The most widely associated genera associated with T2D include *Roseburia, Akkermansia, Faecalibacterium, Bacteroides* and *Bifidobacterium* and are associated negatively with T2D. The bacterial genera having a positive association with T2D include *Ruminococcus, Fusobacterium* and *Blautia* (Gurung et al., 2020). Analysis of stool samples as a part of the South East Asia Community Observatory (SEACO) revealed that Indians have a higher presence of *Clostridiales.* The presence of this genus is inversely associated with T2D in Indians (Larsen et al., 2010). The Uygurs and Kazaks are the two minority groups residing in North-West China. The study revealed that *Ruminococcaceae, Lachnospiraceae* and *Enterobacteriaceae* were predominantly present in all the study participants. The genera of *Planococcaceae* and *Coriobacteriaceae* were significantly elevated in Kazaks without T2D. Whereas, *Veillonellaceae* were significantly abundant in Kazaks with T2D. The genera of *Erysipelotrichaceae* were relatively low in Uygurs with T2D when compared with Uygurs subjects without T2D (Wang et al., 2017). An inter-ethnic study was performed to understand the influence of diet on gut microbial composition. The faecal microbiome was compared between European children and children belonging to village of Burkina Faso in Africa. High throughput 16 S rDNA sequencing analysis revealed that children from Burkina Faso had adequate presence of *Bacteroidetes* and depleted *Firmicutes*. This reflected in presence of abundant SFAs in Burkina Faso children when compared with the European children. This study showed the influence of rural diet and westernized diet on the gut microbiome composition (De Filippo et al., 2010). A multi ethnic study involving non-Hispanic, African-American and Hispanic, White participants and American Indians revealed that African-Americans had abundant *Firmicutes* than Whites. The ratio of Firmicutes/Bacteroidetes was also high in African-Americans. In line with the microbial diversity the levels of acetate and butyrate were significantly low in all African-Americans when compared with the other groups (Hester et al., 2015). Healthy Life in an Urban Setting (HELIUS) study involving Dutch, Ghanaian, Moroccan, African-Surinamese, Turk and South-Asian Surinamese participants was performed to understand the ethnic contributions for gut microbiome. The study revealed that *Prevotella* is predominantly attributed to Moroccans, Turks and Ghanaians. *Bacteroides* were associated with African-Surinamese and South-Asian Surinamese. Dutch participants had rich amount of *Clostridiales.* This study reflected the effect of ethnic differences on people residing in the same geography (Deschasaux et al., 2018).

## Discussion

In this present review manuscript, we explored serum/plasma metabolomics data of T2D subjects in different populations reported in various studies and identified metabolites/metabolic pathways uniquely altered among ethnic groups. Further, we discussed that these metabolic differences may be due to genetic composition along with specific dietary intake reflecting in gut microbiome content among ethnicities. However, this is an observational study and no statistical and bioinformatics analysis was performed due to the unavailability of raw data for T2D metabolomics studies from different ethnic groups. Metabolomics data from diverse studies that we analysed were obtained from different platforms such as LC-MS, GC-MS and NMR and these techniques possess limitations on biochemical analysis of metabolites. Studies conducted to compare metabolic differences in different ethnicities had varied sample size and majority of studies were cross-sectional and case-control studies. The analysis did not consider extrinsic and intrinsic parameters such as BMI, gender, diet, lifestyle and ethnicity. Comparative metabolomics analysis among different populations of T2D, cited in this manuscript were performed in the non-natives from different geographical regions adapted to the newer environment which may mask effects of natural habitats/dietary patterns and lifestyle. Hence, an appropriate study may involve recruiting subjects from their natives and perform comparative metabolomics analysis in different ethnicities using one single platform as a consortium study.

T2D is a complex metabolic disorder characterized by hyperglycaemia, insulin resistance or impaired insulin secretion or both. The 2021 reports of the International Diabetes Federation reveals 537 million adults (20–79 years) living with diabetes across the globe and these numbers may reach 783 million by 2045. The reports also state that three in four adults living with diabetes are from low-and middle-income countries. Epidemiological data indicates geographical differences in prevalence of T2D across the globe (*International Diabetes Federation. IDF Diabetes Atlas, 10th edn. Brussels, Belgium*: International Diabetes Federation 2021). Similarly, shreds of evidence cited in this review demonstrates a significant genetic diversity across ethnic groups along with varied dietary patterns and life style may contribute to altered metabolism in T2D (Ali, 2013), (Scott et al., 2017), (Tabassum et al., 2013). Large scale trials have shown the effect of dietary interventions in the management of T2D and its associated complications (Malik et al., 2010), (Lopez-Garcia et al., 2005), (Mozaffarian, 2016). This suggests multiple metabolic pathways may contribute to manifestation of hyperglycaemia during T2D among ethnicities with different genetic constitution following unique dietary patterns. However, precise supplementation of the missing metabolites boosted the glucose tolerance in certain ethnic groups but not all (Cubbon et al., 2014). Hence, identifying the vital metabolites altered during T2D or at the baseline unique to ethnicities may facilitate in prescribing tailored nutritional therapy/dietary regimen.

## Conclusions

T2D is a systems disease and multiple organs, processes and regulatory pathways are involved in the pathogenesis of T2D. Over the years, inter-ethnic disparities have been demonstrated in both manifestation of T2D and associated co-morbidities such as insulin resistance, adiposity, response to drugs. Hence, comprehensive studies are required such as to (a) identify metabolite correlates of extrinsic and intrinsic factors in large scale metabolomics analysis among various ethnicities and (b) prospective studies in individual patients over the time to get mechanistic insights to understand metabolic changes in T2D subjects during the progression of the disease to vascular complications. Moreover, it is known that the extent to which these metabolic processes may (dys)function differs between patients and hence, T2D must be treated as a self-management disease, focusing on prevention and cure instead of fighting symptoms. Identifying the *‘at risk’* ethnic groups may enable early management of T2D which may serve as better approach to facilitate management of T2D. Ethnicity specific panel of biomarkers may developed for diagnosis, developing therapeutic strategies, suggesting modified diet and prognosis of T2D and associated complications.

## Data Availability

Not applicable.
